# Cell Block as a Surrogate for Programmed Death-Ligand 1 Staining Testing in Patients of Non-Small Cell Lung Cancer

**DOI:** 10.7150/jca.35810

**Published:** 2020-01-01

**Authors:** Zhengwei Dong, Yiwei Liu, Tao Jiang, Likun Hou, Fengying Wu, Guanghui Gao, Xuefei Li, Chao Zhao, Yan Wang, Shuo Yang, Shiqi Mao, Qian Liu, Yumei Li, Chuan Xu, Chunyan Wu, Shengxiang Ren, Caicun Zhou, Jun Zhang, Fred R. Hirsch

**Affiliations:** 1 Department of Pathology, Shanghai Pulmonary Hospital, Tongji University School of Medicine, 200433, Shanghai, P.R. China; 2 Department of Medical Oncology,; 3 Department of Lung Cancer and Immunology,; 4 Department of Oncology and Nursing,; 5 Department of Oncology, Sichuan Academy of Medical Sciences, Sichuan Provincial People's Hospital, University of Electronic Science and Technology of China, Chengdu, Sichuan, 610072, PR China.; 6 Division of Hematology, Oncology and Blood & Marrow Transplantation, Department of Internal Medicine, Holden Comprehensive Cancer Center, University of Iowa Carver College of Medicine, Iowa City, IA, USA; 7 Department of Medicine, Division of Medical Oncology, University of Colorado Cancer Center, Anschutz Medical Campus, Aurora, CO, USA; 8 Clinical Institute for Lung Cancer, Mount Sinai Cancer, Mount Sinai Health System, Tisch Cancer Institute, Icahn School of Medicine, New York, New York.

**Keywords:** NSCLC, PD-L1 expression, cell block, concordance, cytology

## Abstract

**Introduction:** Programmed death-ligand 1 (PD-L1) staining is used in clinical practice to guide the proper use of immune checkpoint inhibitors. This study aimed to investigate the accuracy of PD-L1 staining of non-small cell lung cancer (NSCLC) cytological cell block samples.

**Methods:** Paired cytological cell block and surgical resection samples were consecutively collected from January 2016 to February 2017 in Shanghai Pulmonary Hospital, Tongji University. Two trial-validated PD-L1 assays (28-8 and SP142) were used to quantify PD-L1 expression.

**Results:** A total of 112 pairs of specimens were collected, including 68(60.7%) adenocarcinomas and 28(25.0%) squamous cell carcinomas. Based on a tumor proportion score (TPS) cutoff of 1% for the 28-8 and SP142 assays, PD-L1 expression was positive in 78.6% and 58.9% of surgical samples respectively, while PD-L1 expression was positive in 67.9% and 25.0% of cytological cell block samples.

Based on staining by each antibody, fair to substantial concordance of PD-L1 expression was observed for cytological cell block specimens as compared to surgical resection (𝛋 ranges from 0.377 to 0.686). However, as the tumor cells in the cell block specimen increased, the consistency of PD-L1 expression increased. The concordance of PD-L1 expression in cell blocks with abundant cellularity was nearly perfect with various cutoffs (28-8: tumor cells over 400; SP142: tumor cells over 500).

**Conclusion:** Cytological cell block specimens may serve as a surrogate for PD-L1 staining in patients of NSCLC when more than 400-500 cancer cells were contained (over 400 cancer cells for 28-8, over 500 cancer cells for SP142).

## Introduction

Lung cancer continues to be the deadliest type of malignant tumor worldwide 1. Recently, great progress has been made in understanding the tumor immune microenvironment and gives rise to the development of immunotherapy. Monoclonal antibodies against programmed death 1 (PD1)/ programmed death-ligand 1 (PD-L1) were shown to possess remarkable antitumor activity against several cancers, including non-small cell lung cancer (NSCLC). The efficacy of PD1/PD-L1 inhibitors, such as nivolumab, pembrolizumab, or atezolizumab, has been shown to be superior to that of docetaxel in a second-line setting for patients with advanced NSCLC 2-5. Moreover, in patients with PD-L1 expression tumor proportion score (TPS) ≥ 50%, first-line setting of pembrolizumab has showed superior effects as compared to platinum-doublet therapy^6^. Therefore, detection of PD-L1 expression is important to guide the correct use of immunotherapy in clinical settings.

The availability of adequate amounts of tumor tissue is a challenge for PD-L1 staining in clinical settings 7, similar to testing for mutations in cancer driver genes. Currently, PD-L1 testing is only certified for tissue core biopsies. Since the majority of NSCLC patients are initially diagnosed at an advanced stage, a small biopsy or cytological tumor samples are typically obtained for histological typing and biomarker evaluation, which is a barrier to PD-L1 testing 8. Regarding intratumor heterogeneity^9^, ^10^, PD-L1 testing of cytological cell block specimens should be performed with caution. To date, the evidence of PD-L1 on cell block is scant; therefore, the feasibility of cell block remains controversial. In this study, we retrospectively collected paired surgical and cytological cell block samples in order to investigate the feasibility of performing PD-L1 staining on cell blocks from NSCLC patients and investigate the accuracy of this approach for determining PD-L1 status.

## Methods

### Patients and samples

Patients with operable NSCLC which underwent primary tumor biopsies and surgical resection were collected, at the Shanghai Pulmonary Hospital, Tongji University from January 2016 to February 2017. Patients who have not received systemic therapy or radiation were enrolled. Paired cytological cell blocks and surgical samples was collected. 4-μm sections were obtained from paired FFPE tissue and cell blocks. This study was approved by the ethic committee of Shanghai Pulmonary Hospital, Tongji University and consents were obtained from each patient.

### Cytology sample processing and tumor cell counting

The procedure for cytological cell block processing was similar as described previously 11, 12. Simply, samples were placed into ThinPrep (HOLOGIC Gen-Probe, San Diego, CA, USA) cytology test (TCT) preservation solution. Samples were centrifuged at speed of 2000rpm for 5 minutes. Supernatant was removed and the precipitant was collected. The sediment was packaged by warm agarose gel and had routinely dehydration before packaging in paraffin wax. Then sediment was processed to be embedding and sliced into 4-μm for further staining. Slides for PD-L1 and H&E staining were performed on serial sections from the same specimen. H&E stained slide was used for counting the number of tumor cell on cell block, which was manually counted by two pathologists (Z.D., C.W.) on each cell block via light microscope at 100 magnification. 10 tumor cells were considered as a cluster, then pathologists counted how many clusters contained in each slide of cell block slide.

### Immunohistochemistry procedure

PD-L1 expression was stained with two anti- human PD-L1 rabbit monoclonal antibodies (clone 28-8, ab58810, Abcam, Cambridge, UK; clone SP142; Ventana, Roche Group, Tucson, AZ) using a concentration at 1:60 respectively. After the recovery of antigen bubbled up in EDTA (Ethylene Diamine Tetraacetic Acid) for 8 min and inhibition of endogenous peroxidase activity for 30 min with 3% H_2_O_2_, the sections were incubated in primary antibody for 1 hour at RT (room temperature). Then sections were embedded in second antibody, an HRP Rabbit/Mouse immunoglobulins (Dako, Carpinteria, CA) for 1 hour. The details were described previously^13^, ^14^.

### PD-L1 immunohistochemical evaluation

IHC staining was evaluated independently by 2 pathologists who were blinded to clinical data. If disagreement happened, the third pathologist reviewed the slide and then had a discuss to reach a consensus. TPS was used to categorize the specimens based on multiple cutoff values (1% and 50%).

### Statistical analysis

SPSS software system (version 21.0, SPSS, Inc., Chicago, IL) was used to perform the statistical analysis. The difference between categorical factors and PD-L1 expression, and difference of PD-L1 positive rate in cell block/resected tumor pairs were assessed by chi-square test or Fisher's exact test. The concordance of PD-L1 expression between cell blocks and matching resected tumors were calculated by Cohen's κ coefficient of agreement. Cohen's κ coefficient: the κ value ranged from -1 to 1, with -1 indicating perfect disagreement and 1 indicating perfect agreement. The strength of this agreement is defined as: poor if κ < 0.00; slight if κ was within 0.00 and 0.20; fair if κ was within 0.21 and 0.40; moderate if κ was within 0.41 and 0.60; substantial if κ was within 0.61 and 0.80; and almost perfect if κ was within 0.81 and 1.00. For the subgroup analysis based on tumor cell count, tumor cell count of cell block was used to categorize the cases into subgroups. The alpha for all tests was set at 0.05, therefore, the result was considered statistically significant if p < 0.05. All the tests were two-sided.

## Results

### Patients characteristic

A total of 112 NSCLC patients were included in this study. Among all patients, 79 cases (70.5%) were male, and the median age was 63.9 years old. The majority of patients had stage I NSCLC (59/112,52.7%) and adenocarcinoma (68/112, 60.7%). The majority of cytological cell block samples (102/112, 91.0%) were acquired from computed tomography-guided transthoracic needle aspiration (CT-TTNA), 8(7.2%) cases acquired from endobronchial ultrasound-guided transbronchial needle aspiration and 2(1.8%) were with a bronchoscope brush. The number of tumor cells in the cell block ranged from 10 to 1,500 (shown in the Table 1). 72(64.3%) cell blocks contained between 100 and 1,000 tumor cells, 33(29.5%) cell blocks contained less than 100 tumor cells, and 7(6.3%) cell blocks contained greater than 1,000 tumor cells. More details were seen in Table 1.

### Relationship between PD-L1 staining of cell blocks and pathological characteristics

**Figure 1:**
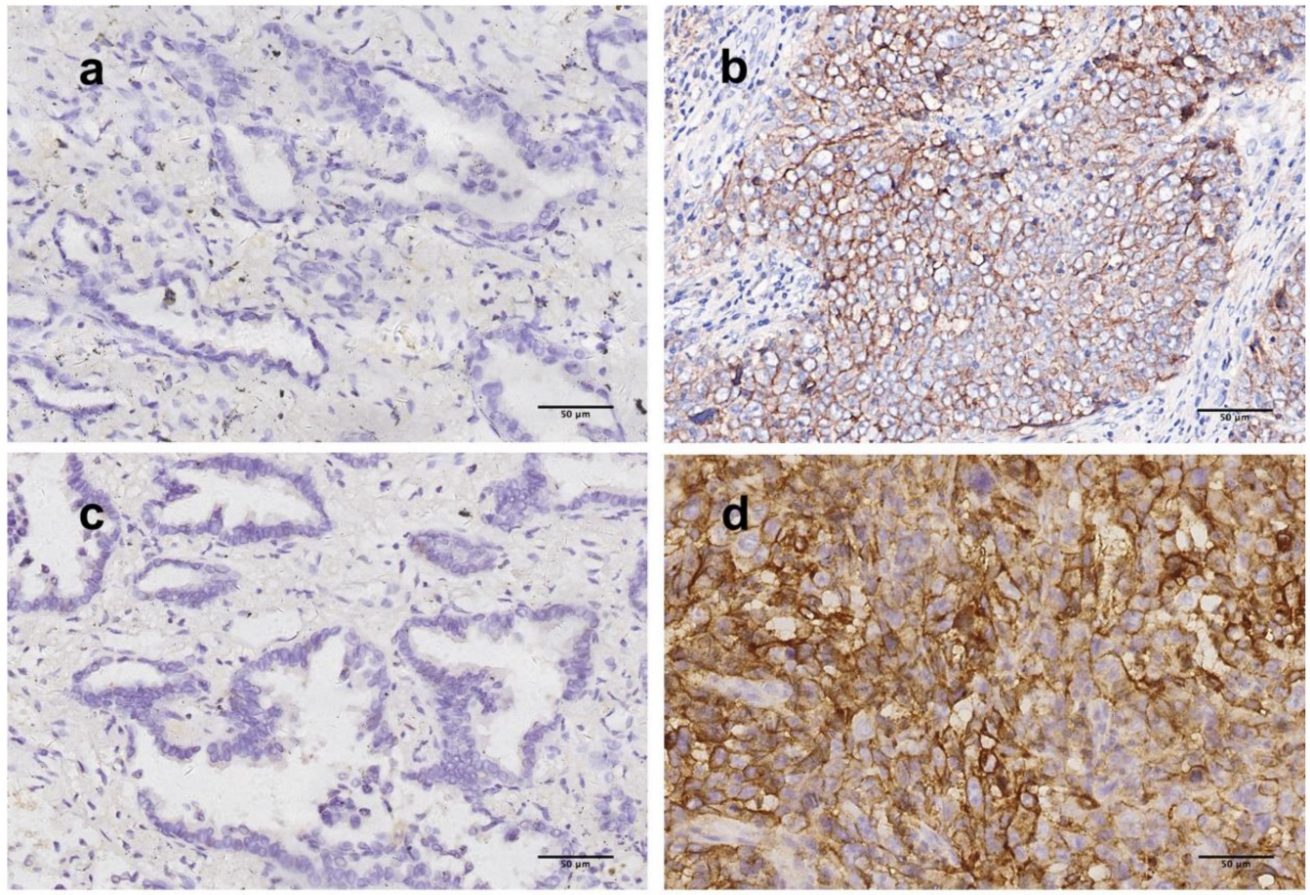
** Representative figures of PD-L1 staining by 28-8 and SP142 assays in resected tumor.** (a, b) Tumor cells stained with 28-8 antibody (200x, original magnification), (a) PD-L1 TPS<1%, (b) PD-L1 TPS>50%; (c, d) Tumor cells stained with SP142 antibody (200x, original magnification) (c) PD-L1 TPS<1%, (d) PD-L1 TPS>50%. PD-L1, programmed death-ligand 1, TPS, tumor proportion score.

**Figure 2:**
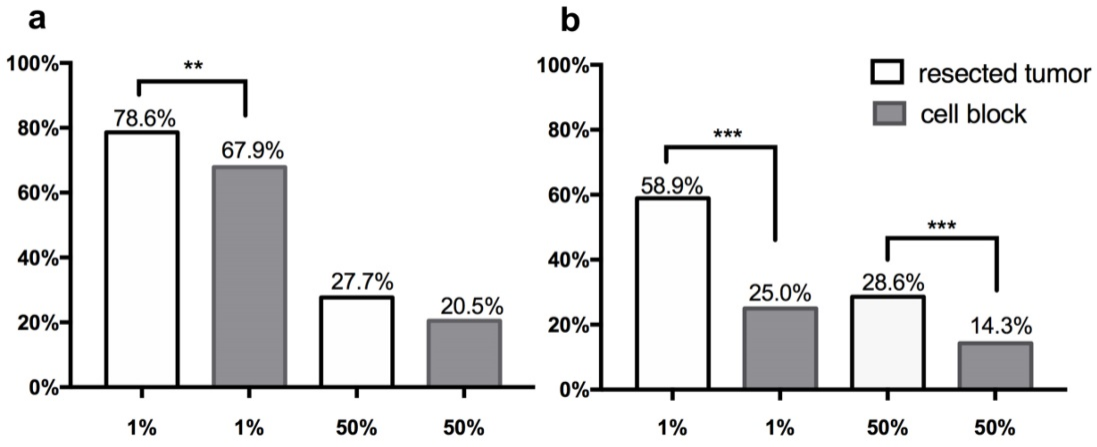
** Frequency of PD-L1 expression on tumor cells of cytological cell blocks and matching surgical specimens with various TPS score cutoffs (1%, 50%) in 112 NSCLC cohort.** Prevalence of PD-L1 stated in vertical axis, and cutoff values for identifying positive stated in the horizontal axis. (a) PD-L1 expression stained by 28-8 assay, (b) PD-L1 expression stained by SP142 assay. p values were determined by chi-square test or Fisher's exact test. * p<0.05, **p<0.005, *** p<0.001.

PD-L1 scoring was performed with two assays (28-8 and SP142). Predominantly membranous staining was observed among 112 tumor/cell block pairs, similar to IHC staining of PD-L1 in previous studies. Representative section of PD-L1 expressing tumor detected by two assays were shown in Figure 1. Two cutoffs (1%, 50%) were used to categorize PD-L1 expression into positive and negative groups. The frequency of PD-L1 expression was shown in the Figure 2.

Based on staining with the PD-L1 28-8 assay, the overall prevalence of PD-L1 expression in tumor cells of the cell block cohort was 67.9% and 20.5%, with cutoffs at 1% and 50% respectively. The rate of PD-L1 positivity was 78.6% and 27.7% in matching surgical sections with cutoffs at 1% and 50%, respectively. PD-L1 expression on surgical sections exhibited a significantly higher frequency with a TPS score≥1%, and a slightly higher frequency with TPS score ≥50% than that on cell blocks.

Based on staining with the SP142 assay, PD-L1 expression was detected in 25.0% and 14.3% of the cases with a TPS of at least 1% and 50% in the cell block cohort. PD-L1 was positive in 58.9% and 28.6% of the cases in the paired resected tumor cohort. The rates of PD-L1 expression with all TPS cutoffs were significantly higher in cytology cell block specimens as compared to surgical specimens (all p<0.001).

### Comparison of PD-L1 between surgical and cytological cell block samples

**Figure 3:**
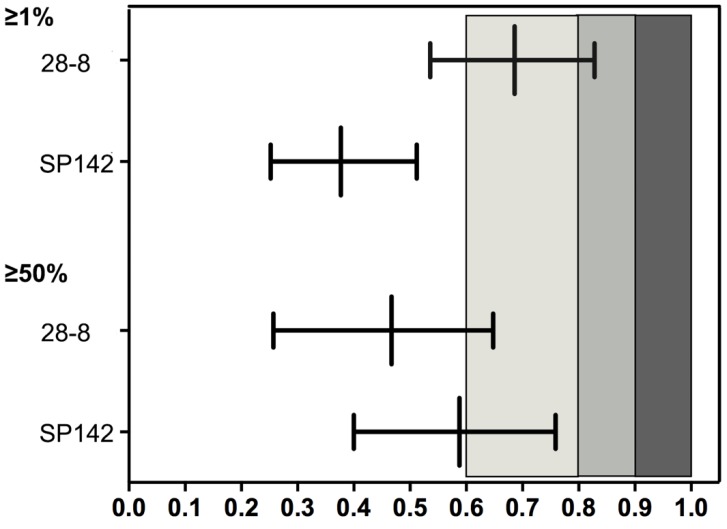
** Reliability of PD-L1 expression between cytological cell block and surgical tumor on tumor cell scoring (presented by 𝜿, ranged by 95% CI).** PD-L1, programmed death-ligand 1; 𝜿, Cohen's κ coefficient. κ value range from -1 to +1, with -1 indicating perfect disagreement and +1 indicating perfect agreement. The strength of this agreement is defined as: poor if κ < 0.00; slight if κ was within 0.00-0.20; fair if κ was within 0.21-0.40; moderate if κ was within 0.41-0.60; substantial if κ was within 0.61-0.80; and almost perfect if κ was within 0.81-1.00.

**Figure 4:**
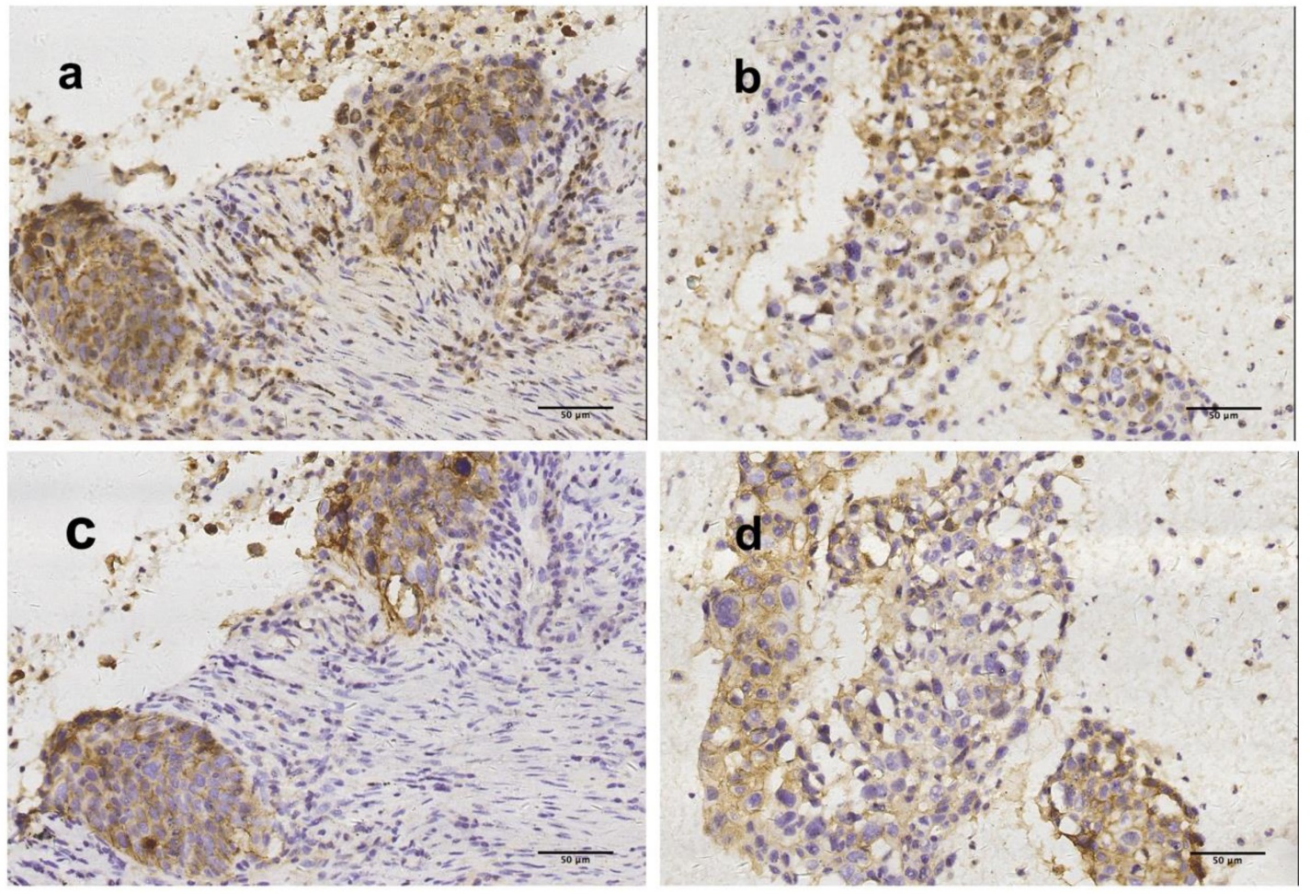
** Representative figures of concordantly positive PD-L1 expression by using surgical tumor tissue and cytological cell block (200x, original magnification).** PD-L1 TPS were >50% (a) and >50% (b) by 28-8 assay in surgical tumor tissue and cytological cell block respectively. PD-L1 TPS were >50% (c) and >50% (d) by SP142 assay in surgical tumor tissue and cytological cell block respectively.

Expression of PD-L1 in cell blocks displayed fair to substantial concordance at all TPS cutoff values when compared with matching resected tumors (Figure 3). A representative section of one PD-L1 TPS concordant tumor from cell block/resected tumor pairs are shown in Figure 4.

Results of PD-L1 staining with 28-8 showed that, 98(87.5%) cell blocks were concordant with matching resected tumors, yielding a κ value of 0.686 (substantial agreement) with a TPS cutoff of at least 1% (reported in the Figure 3). When using PD-L1 expression in resected samples as standard procedure, the sensitivity and specificity of PD-L1 expression on cell block were 85.2% and 95.8%, respectively (shown in the Table S1). 90 cases (80.4%) were concordant, yielding a κ value of 0.467(moderate agreement) with TPS cutoff of at least 50%(reported in the Figure 3). Sensitivity and specificity were 51.6% and 91.4%, respectively (shown in the Table S1).

Results of PD-L1 staining with SP142 showed that 74 of 112 cell blocks (66.1%) matched resected tumors with fair agreement (κ value: 0.377) at a TPS cutoff of at least 1%, and 96 of 112 cell blocks(85.7%) showed consistency with moderate agreement(κ value :0.588) at a TPS cutoff of at least 50% (shown in the Figure 3). Sensitivity and specificity were 42.4% and 100.0% with cut-off at 1%, and 50.0%, 100.0% with cut-of at 50%(presented in the Table S1).

### Correlation between PD-L1 expression and the number of tumor cells in cytological cell blocks

Since the range of tumor cells count(10-1,500) in our study was huge, we further investigated the impact of tumor cells number contained on PD-L1 expression in cell blocks. Subgroup analysis, categorized by division unit, of 50 tumor cells was made. As shown in the Fig. S1, fluctuation of the concordance rate and Cohen 𝛋 value increased as the number of tumor cells increased. Cutoff value for tumor cells were identified as the nearest relative tumor cell number of the fitted curve of κ when κ equaled to 0.80(almost perfect agreement). Therefore, the cutoff value for 28-8 and SP142 was 400 and 500 respectively. For PD-L1 expression measured by staining with 28-8, 96.0% cases were concordant with the TPS cutoff at 1% and 50%, and Cohen 𝛋 values were 0.915 and 0.865 when the tumor cell count reached 400. In addition to concordance, specificity was 100% at all cutoff values. The sensitivity was 100.0% and 80.0% at cutoff value of 1%, 50% respectively. Based on PD-L1 expression as measured by staining with SP142, and 100.0% cases were concordant with Cohen 𝛋 value of 1.000 at cutoff of 50%, when the number of tumor cells reached 500. High specificity (100.0%) and sensitivity (100.0%) was also observed. However, PD-L1 expression as measured by staining with SP142 only showed moderate concordance with 1% cutoff (𝛋:0.468).

## Discussion

This study found that expression of PD-L1 in cytology blocks was inconsistent with resected tumor samples. PD-L1 detection by two companion diagnostic PD-L1 assays showed fair to substantial concordance between cell block/resected tumor pairs. However, PD-L1 scores were more concordant when a greater number of tumor cells were present in the cell block. Cytology cell block specimens were more reliable than surgical resection when enough viable tumor cells were present in the cytological specimens. Therefore, cytology cell block specimens containing sufficient tumor cells could be a surrogate for PD-L1 staining in patients with NSCLC.

The majority of lung cancer patients are initially diagnosed at an advanced stage when resected tumor samples are unavailable 15, 16. In patients with advanced lung cancer, cytological preparations such as fine needle aspiration biopsies or endobronchial ultrasound biopsy specimens are frequently used for pathological or molecular testing 17. However, the feasibility of cytological cell blocks for PD-L1 staining are still unknown. Previous studies with limited samples size showed consistency between cytology cell blocks and matching resected tumor or biopsy samples^18^-^20^. However, BluePrint II study found only moderately good agreement (ICC=0.78-0.85, κ=0.6-0.85) in assessing PD-L1 status on cytological cell block materials^21^. Consistent with this result, our study included 112 pairs of pre-treated resected/cell block tumors between which a fair to substantial level of concordance (66.1% to 87.5%) was found, based on two different PD-L1 assays in various cutoffs. Thus, not all cytology cell block samples are appropriate for PD-L1 expression staining.

Two different mechanisms regulate PD-L1 expression: innate and adaptive expression. Innate PD-L1 expression is induced by aberrant signaling pathways in tumor cells and adaptive expression is induced by exposure to IFN-γ and other chemokines. Therefore, previous studies have found highly geographic heterogeneous PD-L1 staining across different areas of both lung adenocarcinoma and squamous cell carcinoma 22. Also, PD-L1 heterogeneity was found within the tumor by using immunofluorescence to quantify PD-L1 expression^9^. Therefore, heterogeneity of PD-L1 expression limited the amount of tumor tissue that was acquired using small biopsy techniques, which restricted the feasibility of cell blocks to represent PD-L1 expression in the entire tumor. A previous study found a lower positive PD-L1 expression rate and a poor correlation between biopsy samples and the corresponding resected samples, with a discordance rate of 48%^23^. Another study evaluated the inconsistencies of PD-L1 positive cell rate in different microarray cores of adenocarcinoma and squamous cell carcinoma samples in both PD-L1 over-expressed and PD-L1 negative tumors^22^. Similarly, heterogeneity of PD-L1 expression may have also influenced the reliability of PD-L1 scores of cytology cell blocks and may have contributed to fair or substantial concordance between cell blocks and resected samples in this study.

Beside the spatial and temporal heterogeneity of PD-L1 expression, PD-L1 staining heterogeneity might also be a reason interfere reliability. Heterogeneity of PD-L1 staining using different antibodies have been demonstrated in the present study. Pairwise comparisons showed similarity on staining from 22C3, 28-8 and SP263, but the scores from SP142 test showed a significant reduction in labeling of PD-L1 expression in tumor cells^24^, ^25^. This might contribute to an apparent low prevalence of PD-L1 expression stained by SP142 on cell block in our study. Previous studies also showed a lower PD-L1 positivity rate tendency in cytology specimens^19^, ^26^, suggesting that it is still challenging to detect PD-L1 staining by cytological samples and standardization of cytological samples by tumor cell numbers might be a feasible way to perform PD-L1 staining in clinical practice.

Since the tumor cells count had a huge range (10-1,500) in our study, we further investigated the impact of tumor cells number on the PD-L1 expression in cell blocks. We observed a high concordance of PD-L1 expression when cell blocks contained a greater number of viable tumor cells. Similar to our results, previous studies have also shown improved consistency of PD-L1 expression between EBUS-TBNA and biopsy specimens with increased tumor cell count^27^ and cell blocks containing greater than 100 tumor cells would have satisfactory PD-L1 staining results^28^. Our study further investigated the threshold for cell block specimens to serve as a suitable surrogate for PD-L1 staining, which showed that a high rate of concordance was achieved with cutoff of tumor cells set (28-8: TC ≥400; SP142: TC ≥500). In contrast, previous studies demonstrated that cytological samples acquired through EBUS-TBNA or CT-guided fine needle biopsies contained sufficient numbers of tumor cells for histological diagnosis and molecular analysis^29^, ^30^. Therefore, cytological cell block with sufficient tumor cells may be a suitable surrogate for PD-L1 staining in clinical practice.

We should mention that this study had several limitations. First, since it was difficult to obtain paired cytological cell block and surgical resected tumor samples, a small size of 112 paired cases were included in this study. Second, none of the patients were treated with anti-PD1/PD-L1 inhibitors and the predictive role of PD-L1 expression in cell block is still unknown. Third, we mainly collected cell block from primary tumor. However, lymph node sample performed by EBUS/EUS was also an important source for cytological samples. Therefore, further investigation on accordance of cell block on metastatic lymph nodes is needed.

## Conclusions

In conclusion, this study showed the feasibility of staining PD-L1 in cell blocks from NSCLC patients, which is highly consistent with resected specimens when samples contained more than 400~500 cancer cells, suggesting cytological cell blocks may be a suitable surrogate for evaluating PD-L1 expression in cases that have a sufficient number of tumor cells.

## Supplementary Material

Supplementary figures and tables.

## Figures and Tables

**Table 1:** Patient characteristics of the enrolled 112 patients.

Characteristics		N(%) n=112
**Sex**	Male	79(70.5)
	Female	33(29.5)
**Age**	Mean±SD	63.9±8.0
		
**Smoking**	never-smoker	51(45.5)
	former-smoker	1(0.9)
	smoker	60(53.6)
**Gene Mutation**	EGFR	35(31.3)
	EGFR+PI3KCA	1(0.9)
	KRAS	8(7.1)
	ALK	1(0.9)
	ROS1	1(0.9)
	pan-negative	57(50.9)
	unknown	9(8.0)
**Stage**	I	59(52.7)
	II	28(25.0)
	IIIA	22(19.6)
	IIIB	1(0.9)
	IV	2(1.8)
**ECOG PS**	0	16(14.3)
	1	96(85.7)
**Histology**	Adenocarcinoma	68(60.7)
	Squamous cell	28(25.0)
	adenosquamous carcinoma	4(3.6)
	sarcomatoid carcinoma	3(2.7)
	lymphoepithelioma-like carcinoma	2(1.8)
	large cell carcinoma	3(2.7)
	Pulmonary intestinal-type adenocarcinoma	3(2.7)
	Non-small cell carcinoma	1(0.9)
**Type of specimen acquisition**		
	TTNA	102(91.0)
	EBUS-TBNA:	8(7.2)
	Bronchoscope brush	2(1.8)
**Number of tumor cells in the cytology cell block**		
	<100	33(29.5%)
	100~200	27(24.2%)
	200~400	27(24.2%)
	400~500	5(4.5%)
	500~1000	13(11.7%)
	>1000	7(6.3%)
	Median (IQR)	261.5(211.1~316.6)

TTNA: CT-guided transthoracic needle aspiration, EBUS-TBNA: endobronchial ultrasound-guided transbronchial needle aspiration, ECOG PS=Eastern Cooperative Oncology Group performance status. IQR: interquartile range

**Table 2:** Comparison of programmed death-ligand 1 expression between cell block and matching resected tumor.

cut-off	TC Variable		28-8			SP142	
			≥400	<400		≥500	<500
1%	concordance rate		96.0%	85.1%		75.0%	64.1%
	sensitivity		93.8%	83.3%		44.4%	42.1%
	specificity		100.0%	93.3%		100.0%	100.0%
	PPV		100.0%	98.4%		100.0%	100.0%
	NPV		90.0%	53.8%		68.8%	51.5%
	𝛋 value		0.915	0.594		0.468	0.356
50%	concordance rate		96.0%	75.9%		100.0%	82.6%
	sensitivity		80.0%	46.2%		100.0%	44.8%
	specificity		100.0%	88.5%		100.0%	100.0%
	PPV		100.0%	63.2%		100.0%	100.0%
	NPV		95.2%	79.4%		100.0%	79.7%
	𝛋 value		0.865	0.376		1.000	0.527

PPV, positive predictive value; NPV, negative predictive value.
