# Supplementary material for: Cell Block as a Surrogate for Programmed Death-Ligand 1 Staining Testing in Patients of Non-Small Cell Lung Cancer
Source: J Cancer. 2020 Jan 1;11(3):551–8. doi: 10.7150/jca.35810 (PMC6959032; doi:10.7150/jca.35810)

Supplement Table 1: Sensitivity, specificity, PPV and NPV for PD-L1 testing on cell block, when PD-L1 expression on resected tissue was considered as gold standard.

cut-off	Antibody	Sensitivity	Specificity	PPV	NPV
1%	28-8	85.2%	95.8%	98.7%	63.9%
1%	SP142	42.4%	100.0%	100.0%	54.8%
50%	28-8	51.6%	91.4%	69.6%	83.1%
50%	SP142	50.0%	100.0%	100.0%	83.3%

PPV, positive predictive value; NPV, negative predictive value.

Supplement Table 2: Fourfold table for PD-L1 expression status comparison between cell block and surgical tumors, stratified by various cutoffs.

Antibody \ Cutoffs		Cell block					
		1%		sum	50%		sum
28-8		-	+		-	+	
Surgical	-	23	1	24	74	7	81
	+	13	75	88	15	16	31
	sum	36	76	112	89	23	112
SP142		-	+	sum	-	+	sum
Surgical	-	46	0	46	80	0	80
	+	38	28	66	16	16	32
	sum	84	28	112	96	16	112

Supplement Figure 1: Grouped analysis by number of tumor cells on PD-L1 expression in cytological cell block. (A,C) concordance rate of PD-L1 expression stained by 28-8; (B,D) concordance rate of PD-L1 expression stained by SP142.

PD-L1, programmed death-ligand 1; ***κ***, Cohen's κ coefficient. κ value range from -1 to +1, with -1 indicating perfect disagreement and +1 indicating perfect agreement. The strength of this agreement is defined as: poor if κ < 0.00; slight if κ was within 0.00-0.20; fair if κ was within 0.21-0.40; moderate if κ was within 0.41-0.60; substantial if κ was within 0.61-0.80; and almost perfect if κ was within 0.81-1.00.

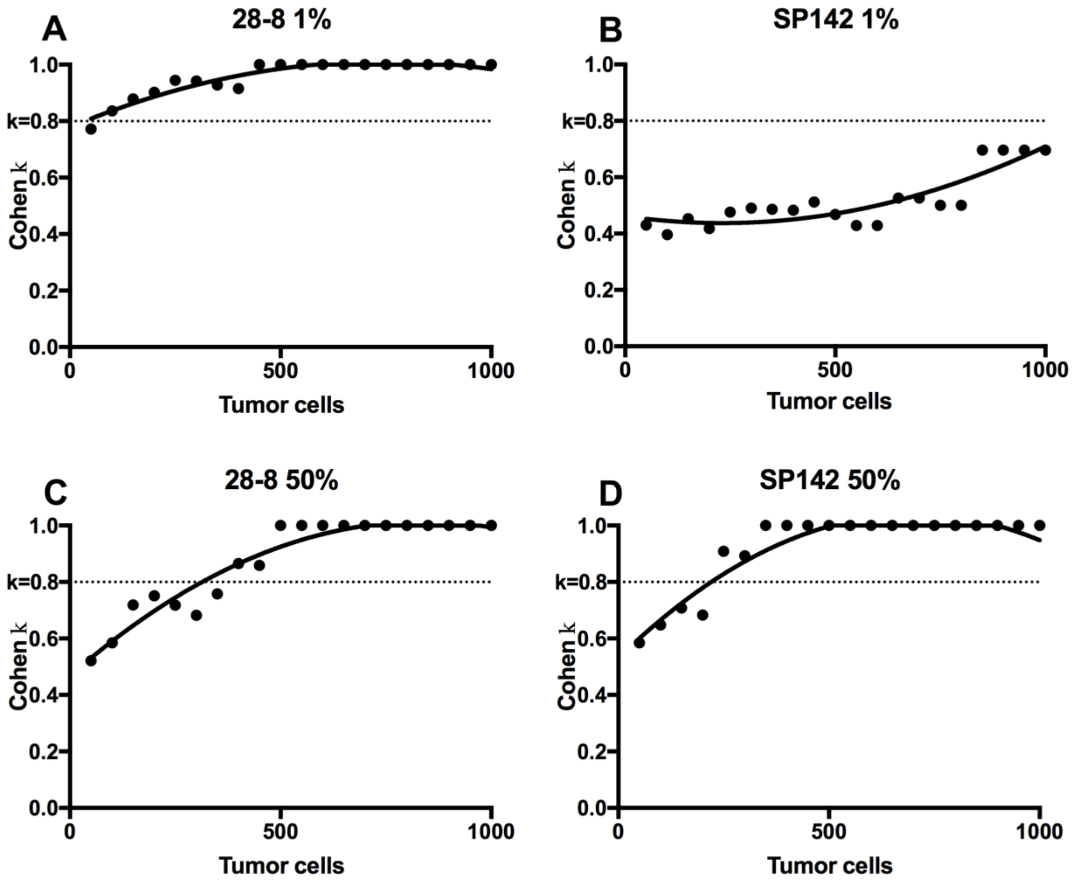

Supplement: Supplementary file 1 — Supplementary figures and tables. [file jcav11p0551s1.pdf]
